# Altered dynamics of glymphatic flow in a mature-onset Tet-off APP mouse model of amyloidosis

**DOI:** 10.1186/s13195-023-01175-z

**Published:** 2023-01-28

**Authors:** Inès R. H. Ben-Nejma, Aneta J. Keliris, Verdi Vanreusel, Peter Ponsaerts, Annemie Van der Linden, Georgios A. Keliris

**Affiliations:** 1 Bio-Imaging Lab, University of Antwerp, Universiteitsplein 1, Wilrijk, 2610 Antwerp, Belgium; 2 Research in Dosimetric Applications, SCK CEN, Boeretang 200, Mol, 2400 Antwerp, Belgium; 3 Laboratory of Experimental Hematology, Vaccine and Infectious Disease Institute (Vaxinfectio), University of Antwerp, Universiteitsplein 1, Wilrijk, 2610 Antwerp, Belgium; 4 μNEURO Research Centre of Excellence, University of Antwerp, Antwerp, Belgium; 5 Institute of Computer Science, Foundation for Research and Technology – Hellas (FORTH), Heraklion, Crete Greece

**Keywords:** Glymphatic system, Brain-fluid circulation, Mature-onset Tet-off mice, Forebrain amyloidosis, Amyloid-beta, DCE-MRI, Astrogliosis, Inflammation

## Abstract

**Background:**

Alzheimer’s disease (AD) is an incurable neurodegenerative disorder characterised by the progressive buildup of toxic amyloid-beta (Aβ) and tau protein aggregates eventually leading to cognitive decline. Recent lines of evidence suggest that an impairment of the glymphatic system (GS), a brain waste clearance pathway, plays a key role in the pathology of AD. Moreover, a relationship between GS function and neuronal network integrity has been strongly implicated. Here, we sought to assess the efficacy of the GS in a transgenic Tet-Off APP mouse model of amyloidosis, in which the expression of mutant APP was delayed until maturity, mimicking features of late-onset AD—the most common form of dementia in humans.

**Methods:**

To evaluate GS function, we used dynamic contrast-enhanced MRI (DCE-MRI) in 14-month-old Tet-Off APP (AD) mice and aged-matched littermate controls. Brain-wide transport of the Gd-DOTA contrast agent was monitored over time after cisterna magna injection. Region-of-interest analysis and computational modelling were used to assess GS dynamics while characterisation of brain tissue abnormalities at the microscale was performed ex vivo by immunohistochemistry.

**Results:**

We observed reduced rostral glymphatic flow and higher accumulation of the contrast agent in areas proximal to the injection side in the AD group. Clustering and subsequent computational modelling of voxel time courses revealed significantly lower influx time constants in AD relative to the controls. Ex vivo evaluation showed abundant amyloid plaque burden in the AD group coinciding with extensive astrogliosis and microgliosis. The neuroinflammatory responses were also found in plaque-devoid regions, potentially impacting brain-fluid circulation.

**Conclusions:**

In a context resembling late-onset AD in humans, we demonstrate the disruption of glymphatic function and particularly a reduction in brain-fluid influx in the AD group. We conjecture that the hindered circulation of cerebrospinal fluid is potentially caused by wide-spread astrogliosis and amyloid-related obstruction of the normal routes of glymphatic flow resulting in redirection towards caudal regions. In sum, our study highlights the translational potential of alternative approaches, such as targeting brain-fluid circulation as potential therapeutic strategies for AD.

**Supplementary Information:**

The online version contains supplementary material available at 10.1186/s13195-023-01175-z.

## Background

Alzheimer’s disease (AD) is an incurable neurodegenerative disorder, clinically identifiable by a gradual cognitive decline, eventually leading to dementia and death [1, 2]. AD is primarily characterised by the accumulation of abnormal protein deposits, namely amyloid-beta (Aβ) and tau. Evidence suggests that protein accumulation coincides with inflammation and the disruption of the neuro-glial-vascular unit function [3–5]. Moreover, the efficacy of debris removal via interacting brain-wide clearance systems such as the recently discovered glymphatic and interconnected brain lymphatic systems is believed to be involved in AD pathology [6–13]. Accumulating evidence suggests a critical involvement of the glymphatic system (GS), which is described as a perivascular network of cerebrospinal fluid (CSF) and interstitial (ISF) fluid exchange facilitated by aquaporin-4 (AQP4) water channels at astrocytic end-feet, in the clearance of Aβ and tau proteins from the brain [8, 14–17]. Furthermore, the CSF movement from the subarachnoid space to the paravascular space was shown to be driven by a combination of factors, such as cardiac and respiratory pulsations, sleep, vasomotion and CSF pressure gradients [14, 18–21].

Importantly, it has been demonstrated that the efficacy of glymphatic transport decreased rapidly upon ageing in wild-type mice [22]. Since ageing represents the highest risk factor for late-onset AD (LOAD), the most prevalent condition in humans (~ 98%), age-related impairments of glymphatic transport could be playing a key role in the progression of AD pathology. However, most of the commonly used transgenic models in AD research were generated based on genetic autosomal dominant mutations of early-onset AD (EOAD; < 65 years old) [23], such as the APP/PS1 and the 5xFAD [24, 25]. One potential drawback these transgenic models have in common is the transgenic gene overexpression or overproduction of Aβ/tau during critical postnatal brain development phases.

Following the discovery of the GS and its implication in AD pathology, several groups have used EOAD models to study glymphatic transport and the factors governing its efficacy in AD. To this end, glymphatic transport was shown to be affected in both young and old APP/PS1 mice compared to wild-type littermates [15]. Furthermore, Xu et al. showed that deletion of AQP4 in APP/PS1 mice worsened Aβ accumulation and memory impairment [26, 27]. In addition, the perivascular localisation of AQP4 channels was postulated to be essential in maintaining the efficacy of the GS, declining with age [22], and related to certain stages of AD pathology [28, 29].

In humans, ultra-fast magnetic resonance encephalography (MREG) imaging at rest demonstrated unique spatiotemporal patterns of low-frequency signals (< 0.01 Hz) associated with dynamics of CSF movement [30]. In addition, CSF dynamics were coupled with global resting-state functional MRI (rsfMRI) signals during non-rapid eye movement (NREM) sleep, implying a neural origin [31]. In line with this, Han and colleagues pointed out a strong coupling between global rsfMRI signals and CSF flow with AD-related pathology including cortical Aβ levels, suggesting a strong link between AD deficits and reductions in brain waste clearance [32]. Moreover, ageing was associated with reduced and more fragmented slow-wave sleep, particularly in AD [33, 34], but also with a decreased CSF flow coinciding with a massive depolarisation of astrocytic AQP4 [22].

Of note, global functional deficits in brain networks as observed using rsfMRI at advanced stages of AD in humans [35, 36] are consistent with measurements in several transgenic EOAD mouse models [37, 38] and the outcome of our recent work where we detected impairments in rsfMRI network integrity in a mature-onset Tet-Off APP mice [39]. In the latter study, APP overexpression was ‘turned-on’ in adulthood (3-month-old mice) when the brain can be considered as mature. This important manipulation ensures that APP overexpression and Aβ overproduction do not occur during the critical period of postnatal development, thereby eliminating false-positive phenotypes unrelated to AD [40]. For instance, the literature indicates that circulating toxic Aβ species have a different impact on neuronal circuits, cell signalling and synapse formation in mature and immature mice [23, 41].

In the current study, we used the same mature-onset Tet-Off APP mouse model as in our previous work on functional neuronal networks [39], to investigate potential changes in glymphatic dynamics at advanced stages of amyloidosis. More specifically, we sought to investigate whether and how the glymphatic transport in Tet-Off APP mice (compared to control age-matched littermates) would be altered in this model with heavy Aβ plaque load in the forebrain. We conjecture that our findings in this LOAD resembling model of amyloidosis can help to dissociate age-related changes from those driven by Aβ pathology and provide valuable information for the future development of treatments against neurodegeneration, brain-fluid circulation dysfunctions and neuroinflammation.

## Methods

### Mouse strain, dox treatment and housing

Generation of the Tet-Off APP transgenic mice has been described in detail in our previous study [39]. Briefly, this inducible model of amyloidosis allows a time-controlled expression of a chimeric mouse/human APP695 transgene using the Tet-Off system. The bigenic tetO-APPswe/ind (line 107) animals (strain B6.Cg-Tg(tetO-APPSwInd)107Dbo/Mmjax, referred to as AD mice in the manuscript) were bred in-house by crossing APP mice, in which a tetracycline-responsive (tetO) promoter drives the expression of the chimeric APP transgene bearing the Swedish and Indiana mutations (mo/huAPP695swe/ind), with transgenic mice expressing the tetracycline transactivator (tTA) gene (strain B6;CBA-Tg(Camk2a-tTA)1Mmay/J). The single transgenic tTA and APP males (Prof. Dr JoAnne McLaurin, Sunnybrook Health Sciences Centre, Toronto, Canada) were initially crossed with non-transgenic females on a C57BL6/J background (Charles River, France) to establish the single transgenic colonies. Since the tTA transgene is under the control of the CaMKIIα promoter, the bigenic mice express APP in a neuron-specific manner at moderate levels, essentially in the forebrain [42]. The APP expression was ‘turned-off’ up to the age of 3 months (3 m, young adult mice) by feeding females with litters and weaned pups with a specific chow supplemented with doxycycline (DOX), a derivative of tetracycline (antibiotics, 100 mg/kg doxycycline diet, Envigo RMS B.V., The Netherlands) from P3 up to 3 months. To induce APP expression in the bigenic mice, all in-house bred transgene carrier and non-transgenic carrier (NTg) littermates were switched to a regular chow from 3 months onward until the day of the surgery (14 months old) resulting in a total APP expression duration of 11 months. Of note, we reused the animals that previously completed a longitudinal rsfMRI study, presented in our recent work [39]. The mouse genotypes are indicated in figure schemes or legends throughout the manuscript. Animals were housed in an environment with controlled temperature and humidity and on a 12-h light–dark cycle, and water was provided ad libitum.

### Surgery

The injection of gadolinium (Gd)-based T1 contrast agent, gadoteric acid (Gd-DOTA), into the cisterna magna (CM) was performed in spontaneously breathing mice anaesthetised with 2% isoflurane (3% for short induction) delivered in oxygen by adapting a previously reported protocol [43, 44]. Briefly, the animal was positioned in a custom-made stereotaxic frame with its head pointing down to expose the CM. A midline incision was made under a microscope from the occipital crest down to the first vertebrae. Then, the underlying muscles were gently separated and maintained pulled aside using two curved forceps. The CM appeared as a small, inverted triangle overlaid with the translucent dural membrane, in between the cerebellum and the medulla. After exposing the CM, 2.5 μl of 50 mM solution of Gd-DOTA (DOTAREM®, Guerbert, France) was injected at 0.55 μl/min via a pulled haematological glass micropipette attached to a nanoinjector (Nanoject II Drummond). To avoid leakage, the micropipette was left in place for an additional 5 min and the incision was closed with biocompatible superglue. The body temperature was maintained at 37.0 °C with a heating pad.

### MRI acquisition

Following surgery, animals were positioned in an MRI-compatible cradle/bed (animal in prone position) using an MRI-compatible mouse stereotactic device, including a nose cone to deliver anaesthetic gas 2% isoflurane (Isoflo®, Abbot Laboratories Ltd., IL, USA) administered in a gaseous mixture of 33% oxygen (200 cc/min) and 67% nitrogen (400 cc/min). During the MRI acquisition, the mice were allowed to breathe spontaneously. The physiological status of the animals was closely monitored during the entire acquisition. The respiration rate was maintained within the normal physiological range (80–120 breaths/min) using a small animal pressure-sensitive pad (MR-compatible Small Animal Monitoring and Gating System, SA instruments, Inc.). The body temperature was monitored by a rectal probe and maintained at 37.0 ± 0.5 °C using a feedback-controlled warm air system (MR-compatible Small Animal Heating System, SA Instruments, Inc.).

**Fig. 1:**
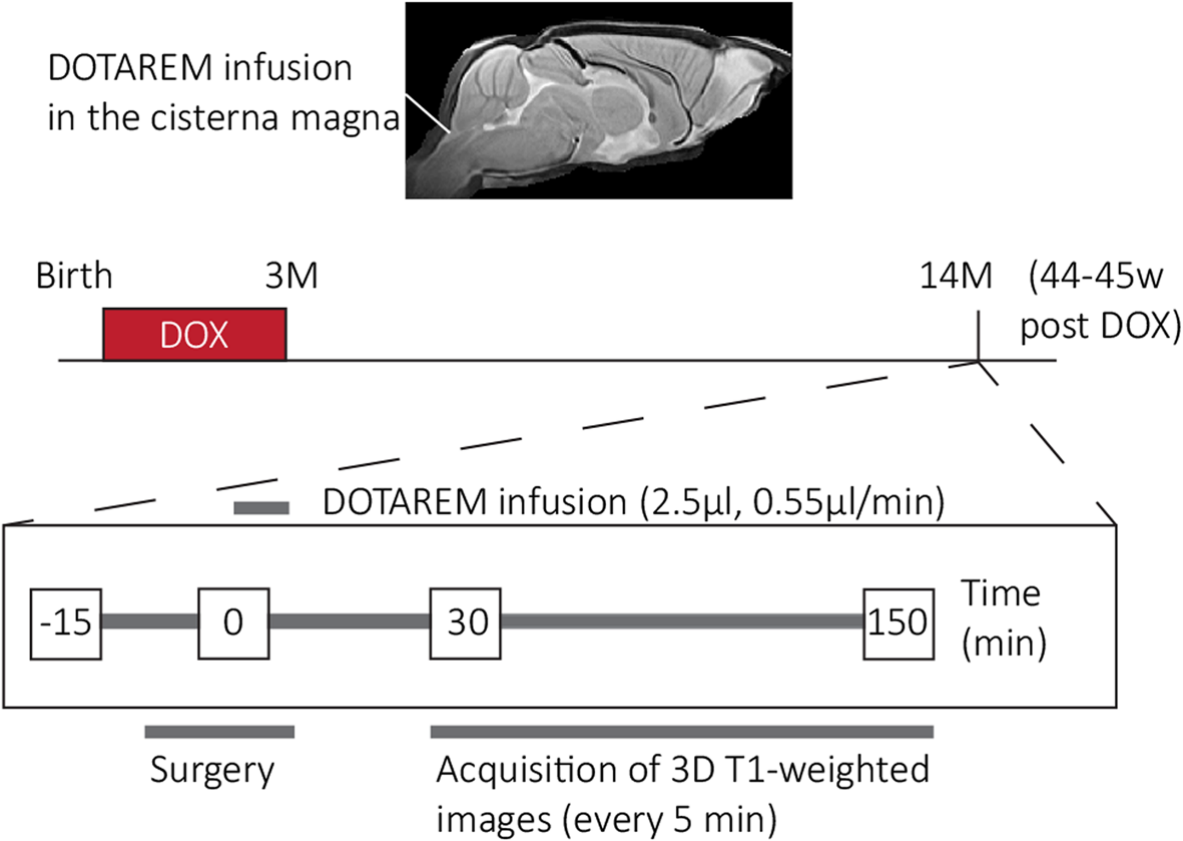
Overview of the experimental setup. The 14-month-old animals, AD and CTL, underwent the surgery after 11 months of amyloid-beta expression. The surgery started a few minutes after the anaesthesia induction (time point =  − 15 min) and the start of continuous Gd-DOTA infusion (2.5 µl, 0.55 µl/min) into the cisterna magna refers to the time point *t* = 0. The DCE-MRI acquisition (120 min) started 30 min post-Gd-DOTA injection, every 5 min, up to 150 min post-injection

All imaging measurements were performed on a 9.4 T Biospec MRI system (Bruker BioSpin, Germany) with the Paravision 6.0 software (www.bruker.com) using a Bruker coil setup with a quadrature volume transmit coil and a 2 × 2 surface array mouse head receiver coil. Axial and sagittal 2D T2-weighted Turbo RARE images were acquired to ensure uniform slice positioning (RARE; TR/TE 2500/33 ms; 9 slices of 0.7 mm; FOV 20 × 20 mm^2^; pixel dimensions 0.078 × 0.078 mm^2^). Dynamic contrast-enhanced MRI (DCE-MRI) acquisition was performed using a 3D T1-weighted FLASH sequence (3D T1-FLASH; TR/TE 15/4.3 ms; flip angle 20°) in the sagittal plane. The field of view (FOV) was 18 × 15 × 12 mm^3^ and the matrix size 96 × 96 × 64, resulting in voxel dimensions of 0.188 × 0.156 × 0.188 mm^3^. The DCE-MRI scans were acquired every 5 min and started 30 min up to 150 min after the contrast agent injection. An overview of the experimental setup is summarised in Fig. 1.

### MRI data pre-processing

Pre-processing of the DCE-MRI data was performed using Advanced Normalisation Tools (ANTs) including realignment, spatial normalisation and creation of a 3D study template. First, a mean image has been created across the time series for each subject and a mask larger than the brain has been delineated on it using AMIRA 5.4. Then, this broad mask has been applied to the DCE-MRI images to remove the surrounding muscle tissue. In parallel, a study-specific 3D template based on the last scan of the non-transgene carrier (NTg) group was created using a global 12-parameter affine transformation followed by a nonlinear deformation protocol. This template was used to estimate the spatial normalisation parameters of the mean images. Next, the realignment parameters of all masked DCE-MRI images within each session to the masked mean image were first estimated, using a symmetric image normalisation method (SyN transformation). Then, the transformation parameters of the realignment and the spatial normalisation were applied to the DCE-MRI images in one resampling step.

Signal intensity normalisation was performed in MATLAB (MATLAB R2020a, The MathWorks Inc. Natick, MA, USA). First, an ellipsoid-shaped region-of-interest (ROI) of 141 voxels was delineated in a cortical area where the variability of the intensity over time was negligible for the baseline and saline groups. More specifically, a time frame of six consecutive scans was selected based on the least changes in the time traces for this specific mask (i.e. intensity values remained approximately constant). Therefore, the mean intensity value of this mask for these six consecutive scans was used to convert each voxel of all images to percent signal change. Finally, a smoothing step was performed with a 3D Gaussian kernel of radius twice the voxel size. A second mask restricted to the brain was applied to all images.

### MRI data analysis

Five groups of 14-month-old mice (14 months) were subjected to DCE-MRI experiments: a non-transgene carrier (NTg) non-injected group (NONE, *N* = 3), a NTg saline-injected group (SAL, *N* = 3), a NTg Gd-DOTA-injected group (CTL_1_; *N* = 5), a tTA Gd-DOTA-injected group (CTL_2_; *N* = 3) and a bigenic Tet-Off APP Gd-DOTA-injected group (AD; *N* = 7). In total, 21 mice (14 months, mixed in gender) were scanned that were reared on the Dox diet until 3 months of age. Five animals (2 NTg, 1 tTA and 2 AD) have been removed due to surgery failure. Given that tTA animals (CTL_2_) do not produce soluble Aβ or Aβ plaques and showed no difference to the NTg littermates (CTL_1_) in our previous resting-state experiments [39, 42], these two groups of animals were combined and are further referred to as the control group (CTL, *N*_CTL_ = 5).

First, a principal component analysis (PCA) was performed per group on the average of the spatially smoothed and normalised time courses. As more than 99% of the data variability could be explained by the three largest components, we used them to reconstruct PCA-based time courses that effectively reduced high-frequency noise from the data. Subsequently, a hierarchical clustering (ward linkage, maximum 15 clusters) was performed on the reconstructed time courses of all animal groups. This analysis allowed for the identification of clusters of voxels with similar time courses and to observe the patterns across groups. Then, to have a fair comparison in the same voxels, the clusters of either the CTL group or the AD group were used to compare the voxel-averaged time courses of the CTL and AD groups and to assess the dynamics of glymphatic flow based on modelling. To this end, we sub-selected the clusters with at least 100 voxels that showed a difference between the maximum and minimum intensity of more than 10%. This criterion was selected based on the variability observed in the NONE and SAL groups for which the time courses were flat as expected (see the ‘Results’ section). Subsequently, PCA was also performed on a subject-by-subject basis to allow for statistical analysis between the groups (CTL vs. AD). Analyses were performed (a) on six predefined hypothesis-driven regions-of-interest (ROIs) and (b) on the clusters defined based on the group-level PCA of the CTL group as described above. For ROI-based analysis, six relevant ROIs (olfactory bulb, hippocampus, medulla, pons, aqueduct and cerebellum) were delineated with MRIcroGL software (https://www.nitrc.org/projects/mricrogl/) based on the 3rd edition of the Paxinos atlas [45]. Then, for each subject, the mean of the PCA-based time courses over the voxels included in each ROI was extracted and the area under the curve (AUC) was calculated and used for statistical analysis (*t*-test across the two groups). For cluster-based analysis, the mean over the voxels included in each cluster was extracted for each subject separately and then the time courses of each cluster were fitted using a model with two exponentials based on the following formula: $f\left(t\right)={c}_{1}\bullet \left(1-{e}^{-\frac{t}{{\tau }_{\mathrm{in}}}}\right)+{c}_{2}\bullet \left({e}^{-\frac{t}{{\tau }_{\mathrm{out}}}}-1\right)$, with *c*_1_ and *c*_2_ representing gain constants, *τ*_in_ the influx time constant and *τ*_out_ the efflux time constant. The estimated *τ*’s for the influx and efflux for each cluster per subject were then used for statistical comparison (*t*-test) across the two groups.

### Immunohistochemistry

Brain samples were collected directly after the MRI acquisition (*N*_NTg_ = 3; *N*_AD_ = 3) as described previously [39]. Briefly, the mice were deeply anaesthetised with an intraperitoneal injection of 60 mg/kg/BW pentobarbital (Nembutal; Ceva Sante Animale, Brussels, Belgium), followed by a transcardial perfusion with ice-cold PBS, and with 4% paraformaldehyde (Merck Millipore, Merck KGaA, Darmstadt, Germany). Brain samples were afterwards surgically removed and post-fixed in 4% paraformaldehyde for 4 h. Next, the fixed brains were freeze-protected using a sucrose gradient (sucrose, Sigma-Aldrich): 2 h at 5%, 2 h at 10%, and overnight at 20%. Then, the brain samples were snap frozen in liquid nitrogen and stored at − 80 °C. Finally, 14-μm-thickness sagittal brain sections were cut using a cryostat (CryoStar NX70; ThermoScientific).

For immunofluorescence analyses, the following primary antibodies were used: chicken anti-GFAP (Abcam ab4674, 1:1000), rabbit anti-IBA-1 (Wako 019–19,741, 1:1000), rabbit anti-AQP4 (Sigma-Aldrich HPA014784, 1:100) and the following secondary antibodies: donkey anti-chicken (Jackson 703–166-155, 1:1000), goat anti-rabbit (Jackson 111–096-114, 1:1000) and donkey anti-rabbit (Abcam AF555, 1:1000). Moreover, the Aβ plaques were stained with Thioflavin-S (Santa Cruz Biotechnology, sc-215969) and the vessels with lectin (Labconsult VEC.DL-1174 (green) or VEC.DL-1177 (red), 1:200). After the staining, the sections were mounted using Prolong Gold Antifade (P36930; Invitrogen).

Immunofluorescence images of GFAP/lectin, Iba1/lectin, AQP4/lectin and Thioflavin-S/lectin stainings were acquired using an Olympus BX51 fluorescence microscope equipped with an Olympus DP71 digital camera and the image acquisition was done with CellSens Imaging Software (Olympus, Tokyo, Japan, http://www.olympus-global.com). Obtained images were visually evaluated by at least three co-workers to ensure the selection of representative images. The 3rd edition of the mouse brain atlas from Paxinos and Franklin was used as a reference for the localisation of the regions of interest. Images were further processed with ImageJ Software 1.52 k (National Institutes of Health) and artificially pseudo-coloured in the representative images.

Histological quantifications were performed in FIJI version 2.9.0/1.53t. Estimation of the vascular density in the cortex was performed in two-channel immunofluorescence images of Lectin/ThioflavinS. First, the two channels were split and then the Lectin (vascular) channel was auto-thresholded using the Triangle method implemented in FIJI. To eliminate noise, only clusters with greater than 300 pixels were retained. In addition, areas where plaques were present in the ThioflavinS channel were excluded from the analysis. The vascular density was estimated as the fraction of the total area of clusters to the surrounding pixels. Similarly, (a) the total GFAP-positive signal reflecting potential changes in astrogliosis and (b) the fractional vascular coverage by astrocytes were performed using two-channel immunofluorescence images of GFAP/lectin that were also split into two image files for each area (cortex, amygdala, brainstem). For the GFAP analysis, each image was auto-thresholded using the Li method implemented in FIJI and only clusters greater than 50 pixels were retained. The intensity of each of the clusters was then measured and averaged over all clusters to calculate the total mean GFAP signal intensity. For vascular coverage analysis, the image from the corresponding lectin channel was auto-thresholded using the Triangle method in FIJI and only clusters over 300 pixels were retained. Subsequently, to calculate the overlap of astrocytes on vessels, the two thresholded images from the GFAP and lectin channels were merged, converted to RGB composite images and thresholded for colour hues between 10 and 80 (i.e. orange to greenish yellow). The number of pixels in this overlap was divided by the number of pixels in the thresholded lectin channel to calculate the fractional vessel coverage by astrocytes. Vascular density, total GFAP signal intensity and fractional vessel coverage were then used for statistical analysis.

### Statistical analyses

For the MRI ROI-based analysis, a two-sample *t*-test was performed on the area under the curve (AUC) values per ROI for the CTL vs. AD groups. Similarly, for cluster-based analysis, a two-sample *t*-test was performed on the *τ*_in_ and *τ*_out_ values per cluster across the two groups. MRI statistical analyses were performed using MATLAB (version 9.8 (R2020a), Natick, Massachusetts, The Mathworks Inc.). Significance was defined with a criterion *α* = 0.05. All results are shown as mean ± standard errors.

For immunohistochemistry statistical analyses of vascular density, total GFAP signal intensity and fractional vessel coverage, we performed linear mixed-effects (LME) model analyses (*fitlme* function) in MATLAB (version 9.10 (R2021a)). For vessel density (that was only estimated in the cortex), genotype was used as a fixed effect and animal id as a random effect. For total GFAP signal intensity and fractional vessel coverage, genotype and brain area were used as fixed effects and animal id as a random effect. Significance was defined with a criterion *α* = 0.05. Result means are reported with their 95% confidence intervals (CIs). More details of the parameter fit for the LME analysis are reported in the Supplementary Data.

## Results

### Differences in the spatiotemporal distribution of Gd-DOTA in the Tet-Off APP mice vs. controls

**Fig. 2:**
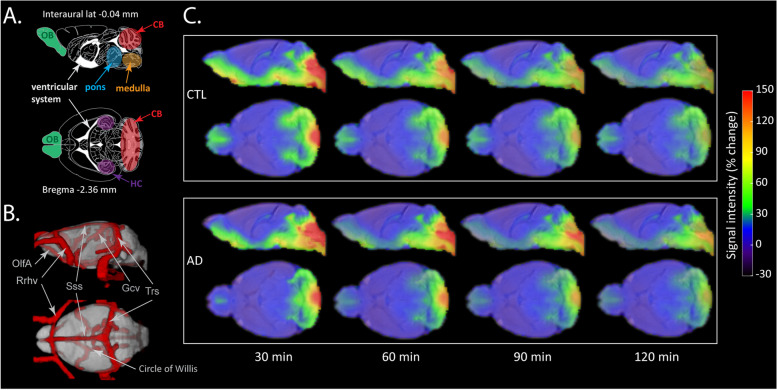
Spatial distribution maps of Gd-DOTA in the AD and CTL mice over time. **A** Schematic representation of the sagittal plane (interaural lateral − 0.04 mm) and transverse plane (Bregma level − 2.36 mm) from the Paxinos atlas with highlighted key regions such as olfactory bulb (OB), cerebellum (CB), hippocampus (HC), pons, medulla and ventricular system. **B** 3D visualisation of major blood vessels based on a recent vascular brain mouse atlas that was aligned to the template of this study (vascular atlas from [46]). The depicted vessels including OlfA (olfactory artery), Rrhv (rostral rhinal vein), Gcv (cerebral vein of Galen), Sss (superior sagittal sinus) and Trs (transverse sinus) are located in the vicinity of regions with enhanced contrast. **C** The spatial distribution of the MRI contrast agent in the CTL (top panel) and AD mice (bottom panel) at 30 min, 60 min, 90 min and 120 min post-intracisternal infusion shown in sagittal and transverse views. The colour scale represents the average percent signal intensity change, with dark blue/red indicating low/high percent signal intensity change, respectively

We first investigated the efficacy of glymphatic transport in 14-month-old Tet-Off APP (AD) and control (CTL) mice by assessing the dynamics and distribution patterns of a contrast agent (Gd-DOTA) in the brain. For this, T1-weighted contrast-enhanced MR images were acquired sequentially from 30 to 150 min following the labelling of CSF upon infusion of Gd-DOTA into the cisterna magna (*t* = 0). We detected clear differences in the dynamics of brain-wide distribution patterns of Gd-DOTA, with a reduced rostral glymphatic flow in the AD group and higher accumulation of contrast agent within caudal regions of the brain (see Fig. 2C for an overview of the Gd-DOTA distributions in the brains of the two groups of animals).

In line with the literature, characteristic patterns of GS-related contrast agent distribution were observed in our CTL mice, with contrast enhancement of the paravascular routes and the adjacent parenchyma [8, 18, 43, 47–49]. More specifically, the CTL mice showed a high accumulation of Gd-DOTA at 30 min post-injection, as reflected by the high signal intensity in the cisterna magna (CM), ventricular system, ventrally along the circle of Willis and olfactory paravascular pathways (Fig. 2A–C, top panel), as well as in caudal parenchymal brain regions including the brainstem (i.e. medulla and pons). Other regions of detected contrast enhancement included the cerebellum (CB), the pituitary recess, the ventral part of the thalamus and the olfactory bulb (OB). In addition, contrast enhancement was also observed in the areas adjacent to the transverse sinuses (see also Additional Fig. 1). Starting from 60 min onward, the signal intensity of the Gd-DOTA started to fade drastically, except for the regions located in the vicinity of the cisterna magna, which continued to demonstrate enhanced contrast.

At the imaging onset (*t* = 30 min), the Gd-DOTA distribution for the AD group was spatially largely similar to the controls, while differences became more pronounced over time (Fig. 2C, bottom panel). Indeed, at 30 min post-infusion, we noticed that the T1 signal intensity was higher at the caudal brain regions, including the ventral part of the cerebellum, medulla and pons, while lower contrast enhancement was observed within the olfactory bulb. From 60 min post-infusion onward, the heterogeneity in spatial differences was evident, with a higher signal intensity of the Gd-DOTA, retained for a longer time in the brainstem (i.e. pons and medulla) and the ventral part of the CB. Compared to the CTL group, a lower contrast enhancement was detected within the OB.

### Caudal retention and reduced rostral flow of Gd-DOTA in AD mice

**Fig. 3:**
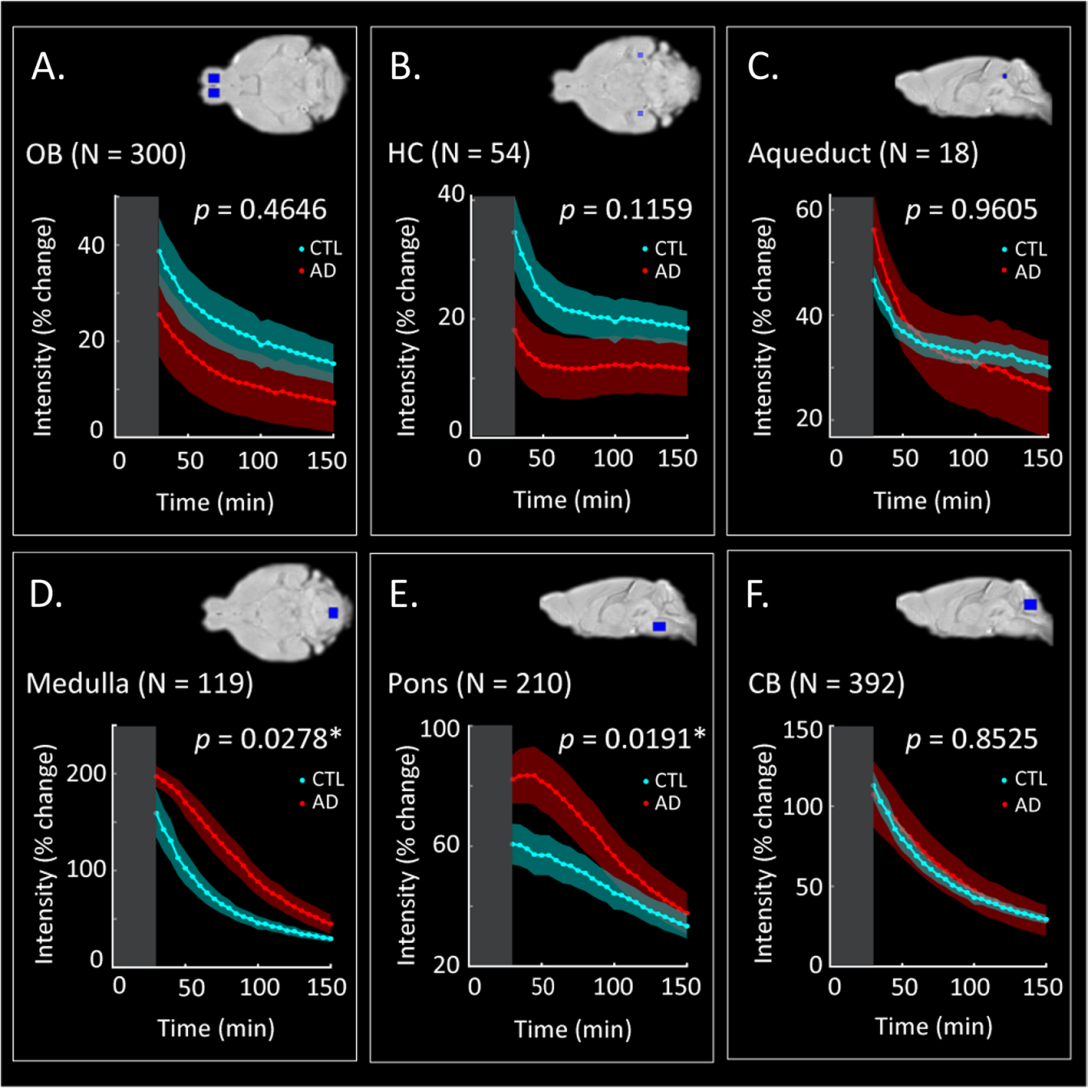
Region-of-interest (ROI)-based analysis. Average signal intensity changes over time reflecting Gd-DOTA contrast agent distribution in the OB (**A**), the HC (**B**), the aqueduct (**C**), the medulla (**D**), the pons (**E**) and the CB (**F**) for the CTL (cyan) and the AD (red) groups of mice. The grey rectangle in each panel represents the pre-acquisition time (acquisition started at 30 min post-Gd-DOTA infusion) with zero indicating the time of injection. An overview of the brain location of each ROI can be found in the top right corner of each panel (blue rectangles). OB, olfactory bulb; HC, hippocampus; CB, cerebellum; N, ROI size in voxels; *significant difference in the area under the curve (AUC) across the two groups indicates a differential distribution of contrast agent (*N* = 5 mice per group; two-sample *t*-test; *p* < 0.05; see the ‘Methods’ section)

To investigate the glymphatic transport in more detail, the quantification of Gd-DOTA accumulation was assessed within specific anatomical regions. To this end, a ROI-based analysis was performed with the outcome being illustrated in Fig. 3. In line with results highlighted in the previous section, the AD mice showed significant differences in signal intensity in the medulla (*p* = 0.0278, Fig. 3D) and the pons (*p* = 0.0191, Fig. 3E) compared to CTL mice, while no differences were found in the OB, CB and aqueduct (Fig. 3A–C, F). Furthermore, a trend for slightly higher accumulation of Gd-DOTA, albeit not statistically significant, within the hippocampus (Fig. 3B, *p* = 0.1159) was found for CTL littermates. Thus, these data indicated that glymphatic circulation was altered in AD mice, and transport of Gd-DOTA from the cisterna magna towards the rostral and dorsal parts of the brain was reduced and accompanied by prolonged accumulation at the caudal regions (medulla and pons) that are free of amyloid plaques [42].

### Clustering of voxel time courses reveals differential Gd-DOTA spatial patterns in AD and CTL mice

**Fig. 4:**
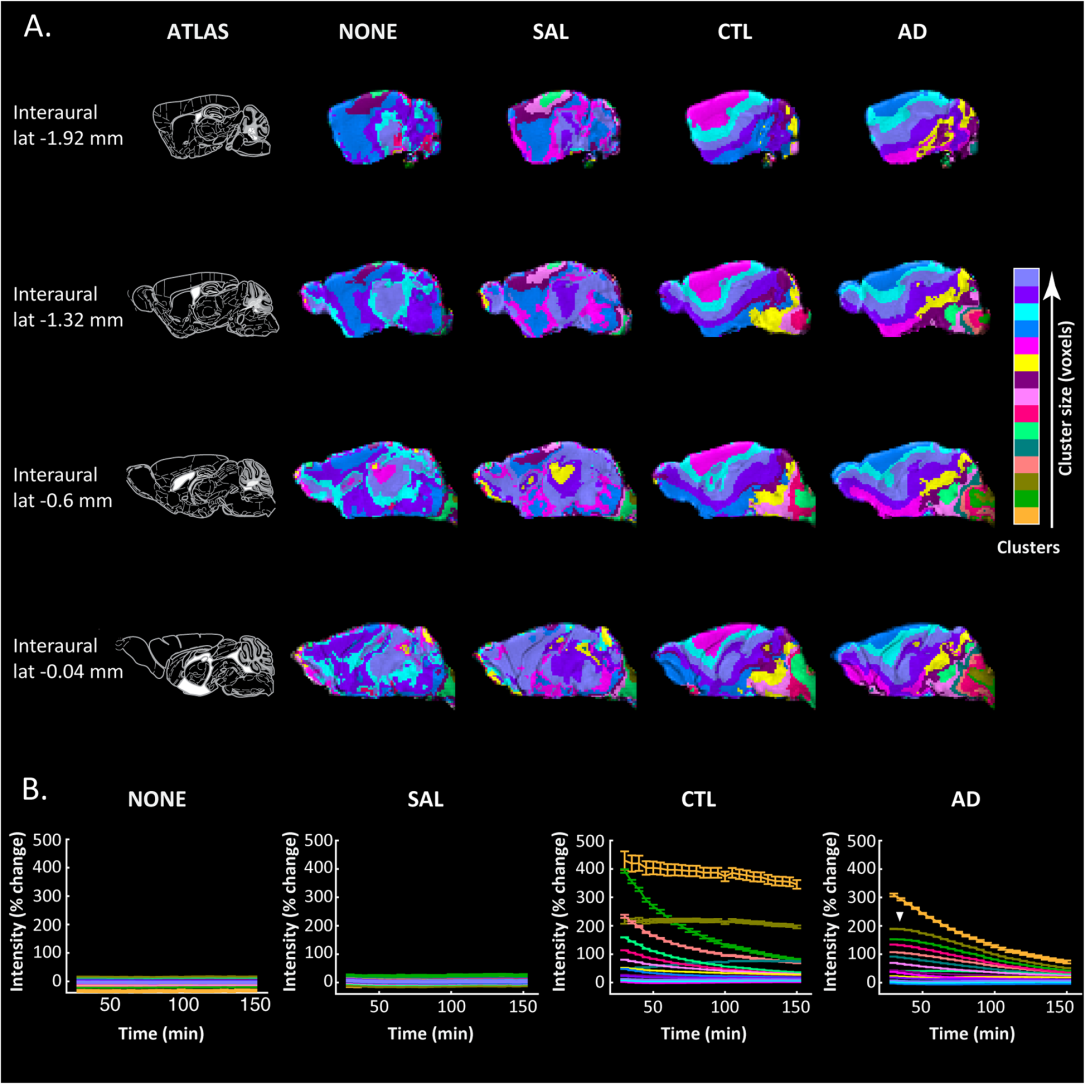
Gd-DOTA spatial patterns over time in the Tet-Off APP mouse model. The clustering of MRI voxel time courses reflecting Gd-DOTA contrast agent distribution over time in the Tet-Off APP (AD) and three control groups: non-injected (NONE), saline-injected (SAL) and Gd-DOTA injected (CTL). **A** On the leftmost column (ATLAS), sagittal planes from the Paxinos atlas at different interaural locations indicate four representative brain slices. In other columns, the visualisation of 15 clusters for each group (each cluster shown with a different colour) demonstrates brain areas that share similar distributions of contrast agent. Colours on the colour bar indicate the sorting of clusters (in number of voxels) from smallest to largest in each group but note that same colours in different groups do not entail the same sizes and that it is not a linear scale but rather a sorted legend. **B** The voxel-average time courses (mean ± SD) of each cluster are shown for each group in the same colour as the respective cluster in **A**. Note that non-injected groups (NONE, SAL) show flat distributions of signal intensity over time that simply relate to slight signal amplitude differences without contrast agent contribution. On the other hand, injected groups demonstrated large increases in signal intensity starting at 30 min post-injection of Gd-DOTA (start of MRI acquisition) declining over time in most of the clusters (clearance of contrast agent) but also noted the accumulation of contrast agent in a couple of clusters

Given the observed differences in the distribution of Gd-DOTA in the selected ROIs over time (Figs. 2 and 3), we sought to evaluate whether the similarity of the time courses of glymphatic transport across the brain could provide information about the distribution patterns and pathways. To this end, we performed cluster analysis on the group average PCA-based time courses by using the three largest principal components that could explain ~ 99% of the data variability. The resulting clustering maps for each group are shown in Fig. 4A and the corresponding time courses in Fig. 4B.

As expected by the absence of a contrast agent, the clustering maps of the non-injected (NONE) and saline-injected (SAL) groups did not show a particular structure, but rather consisted of some very large homogeneous clusters and some smaller ones distributed randomly over the brain (Fig. 4A). Moreover, as shown in Fig. 4B, the time courses for the SAL and NONE groups were approximately constant over time and close to 0% intensity change, essentially showing that the percent signal intensity change over time was minimal and much smaller in relation to the contrast injected groups. The small variability in the intensities of different clusters, approximately ± 5%, was used to guide the criterion for selecting clusters for further analysis in the injected groups, i.e. the clusters showing substantial intensity changes over time and thus reflecting changes in the distribution of contrast agent rather than simple signal variability (see the ‘Methods’ section).

Contrary to the NONE and the SAL groups, the CTL and AD groups that received intracisternal Gd-DOTA injections displayed distinct clustering patterns. We could clearly observe a gradual spreading of the clusters from the posterior of the brain proximal to the cisterna magna towards the anterior of the brain and the olfactory bulb and dorsally towards the cortex (Fig. 4A). Furthermore, we noticed that for the AD group, the clusters proximal to the cisterna magna were more heterogeneous (fragmented) compared to the CTL. In addition, the time courses of some clusters in the AD mice showed an apparent delay in reaching the maximum peak compared to those in the CTL (Fig. 4B; white arrow).

### The dynamics of glymphatic transport are altered in the AD mice

**Fig. 5:**
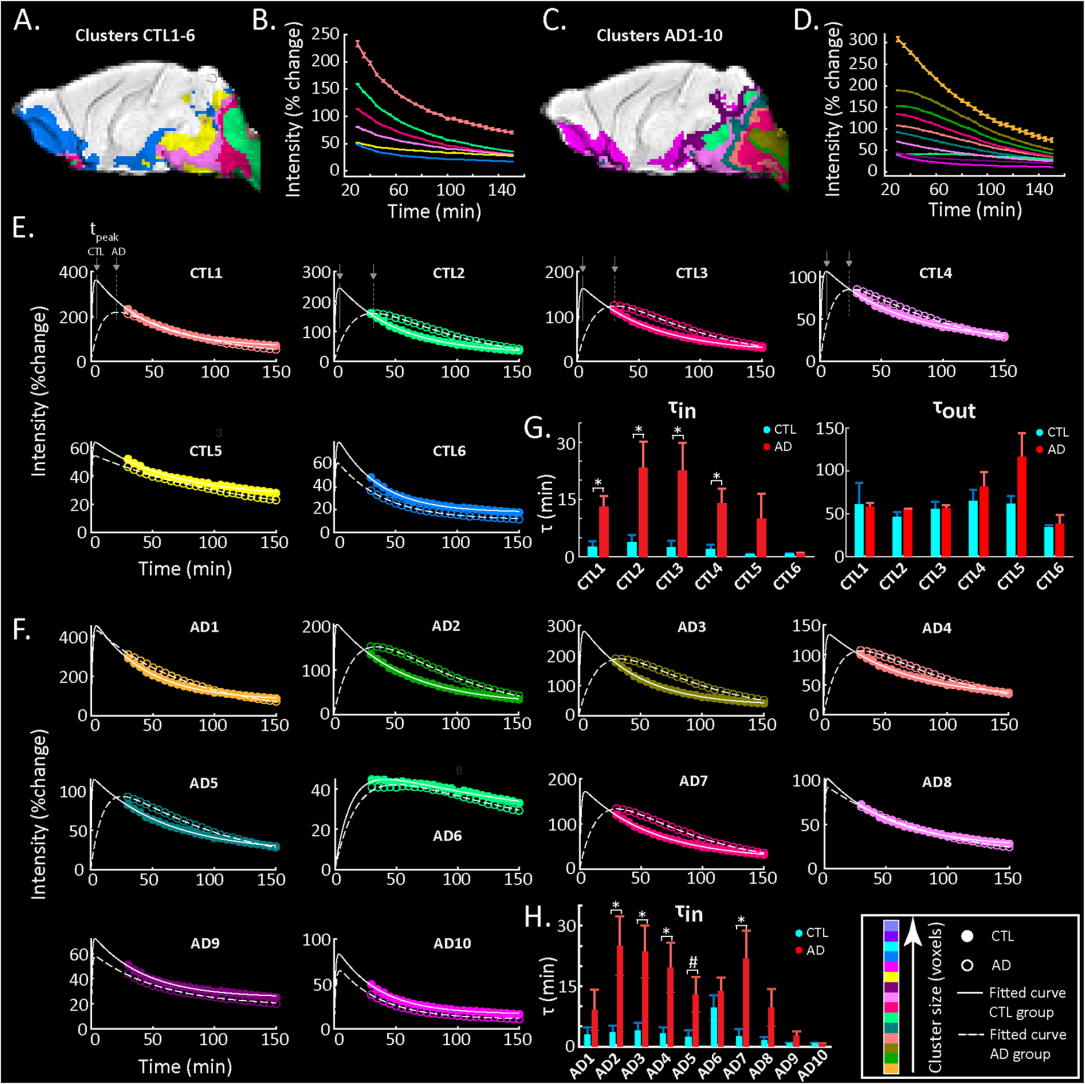
Model fitting of cluster time courses. **A**, **C** Clusters of the CTL and AD groups respectively selected for further analysis based on a minimum signal intensity change and a minimum number of voxel criterion (see the ‘Methods’ section). **B**, **D** Time courses of the selected clusters in the CTL and AD groups respectively. **E** Model fitting of the time courses of the selected clusters from the CTL group (solid lines, filled circles) is compared to model fitting of the same cluster of voxels in the AD group (dashed lines, open circles). **F** Similarly, model fitting of the selected clusters of the AD group (dashed lines, open circles) is compared to model fitting of the same cluster of voxels in the CTL group (solid lines, closed circles). In each panel of **E** and **F**, lines demonstrate the model fit and circles the data points (also shown in **B**, **D**) for each cluster. Colours represent the size sorting of the clusters based on the number of voxels (same as Fig. 4). **G** Bar graphs of influx (*τ*_in_) and efflux (*τ*_out_) time constants for CTL and AD mice in the clusters of the CTL map shown in **A**. Mean values and sem as well as the *p*-values are reported in Table 1. **H** Bar graph of the influx (*τ*_in_) time constant for CTL and AD mice in the clusters of the AD map shown in **C**. Efflux is not shown (no significant differences) but values for *τ*_in_ and *τ*_out_ as well as *p*-values are reported in Table 2. For all bar graphs, ‘*’ denotes statistical significance (*N* = 5 mice per group; two-sample *t*-test; *p* < 0.05)

Based on the obtained clustering maps and time curves, we sought an in-depth comparison between the AD and CTL mice to unravel differences in the dynamics and kinetics of glymphatic transport. To this end, we selected the clusters in each group with (a) a sufficient number of voxels (*N* ≥ 100) and (b) differences in percent signal change over time greater than a threshold of at least 10% (Fig. 5A–D). Note that the selected threshold was chosen based on the percent signal change variability we observed across clusters in the NONE and SAL groups (Fig. 4B). To compare the dynamics of the two groups across the same voxels, we used as a reference either the clusters that passed the selection criteria for the CTL group (*N* = 6; CTL1-6; Fig. 5A, E) or the AD group (*N* = 10; AD1-10; Fig. 5C, F). Next, we extracted the time courses from the same clusters in each animal and fitted them using a double exponential model with parameters representing the time constants for the influx (*τ*_in_) and the efflux (*τ*_out_) of the Gd-DOTA. The group average time courses and the corresponding fitting curves using the clusters from the CTL and AD groups are shown in Fig. 5E (CTL1-6) and F (AD1-10), respectively.

Using as reference the CTL-based clusters (Fig. 5E), we noticed that those located in the vicinity of the injection site showed different profiles of data and fitted curves across the two groups (Fig. 5E; CTL1-4). While the CTL group curves were from the start of acquisition in the descending phase of signal intensity (representing the efflux of the Gd-DOTA), initial increases of signal intensity (representing influx and accumulation of Gd-DOTA) were still occurring in the AD mice with signals reaching the maximum peak at later time points. Furthermore, the infiltration of Gd-DOTA into the brain parenchyma was significantly longer in the AD group. These observations were also consistent with the model fitted *τ*_in_ time constants representing contrast agent influx, which showed significant differences across the two groups (see *τ*_in_ values of clusters CTL1-4 in Table 1). In contrast, two other clusters (CTL5 and CTL6) displayed similar patterns in both groups and no significant differences in the influx were observed. Notably, in the case of the efflux time constant *τ*_out_, we found no significant differences between the AD mice and their CTL littermates for any of the CTL-based clusters (Table 1).

**Table 1:** Influx and efflux time constants of the CTL clusters. *p*-values of the two-sample *t*-test performed on the time constants *τ*_in_ and *τ*_out_ (reported as mean ± sem [min] across animals) representing the influx and efflux of DOTAREM for the CTL-based clusters. *p < 0.05

*Cluster*	CTL1	CTL2	CTL3	CTL4	CTL5	CTL6
*p-value (in)* =	0.0149*	0.0248*	0.0199*	0.0274*	*0.1893*	*0.6197*
*τ*_in (CTL)_ [min]	*2.6* ± *1.5*	*3.9* ± *1.8*	*2.5* ± *1.7*	*2.0* ± *1.2*	*0.7* ± *0.1*	*0.8* ± *0.1*
*τ*_in (AD)_ [min]	*13.1* ± *2.8*	*23.3* ± *6.8*	*22.6* ± *7.2*	*13.9* ± *3.9*	*9.9* ± *6.5*	*0.9* ± *0.1*
*p-value (out)* =	*0.9047*	*0.2088*	*0.4702*	*0.9026*	*0.0955*	*0.6765*
*τ*_out (CTL)_ [min]	*61.2* ± *25.1*	*46.0* ± *5.9*	*55.4* ± *8.3*	*65.1* ± *13.1*	*61.7* ± *9.0*	*34.2* ± *2.4*
*τ*_out (AD)_ [min]	*58.0* ± *5.0*	*54.4* ± *1.8*	*56.5* ± *3.3*	*81.5* ± *17.4*	*116.7* ± *27.7*	*38.6* ± *9.9*

Using as reference the AD-based clusters (Fig. 5F), even though their spatial distribution was different with more clusters in total, a similar effect—namely the influx of Gd-DOTA was significantly slower in the AD mice—was observed particularly for those clusters in the vicinity of the injection site (Fig. 5F; AD2,3,4 & 7). This was also reflected in the statistics of the *τ*_in_ and *τ*_out_ time constants across the two groups (Table 2). Note that AD5, which consisted of voxels surrounding the aforementioned clusters and thus was receiving Gd-DOTA from them, also showed a statistical trend (*p* = 0.0563) for a difference in the influx *τ*_in_ parameter across the two groups. In analogy to the CTL clusters, the efflux *τ*_out_ of Gd-DOTA was similar between both groups with no significant differences in any cluster (see *p*-values of *τ*_out_ in Table 2).

**Table 2:** Influx and efflux time constants of the AD clusters. *p*-values of the two-sample *t*-test performed on the time constants *τ*_in_ and *τ*_out_ (reported as mean ± sem [min] across animals) for the influx and the efflux of the DOTAREM for the AD clusters. *p < 0.05; ^#^trend for statistical significance

*Cluster*	AD1	AD2	AD3	AD4	AD5	AD6	AD7	AD8	AD9	AD10
*p-value (in)* =	*0.2868*	0.0209*	0.0204*	0.0327*	*0.0563*^*#*^	*0.4011*	0.0260*	*0.1161*	*0.1541*	*0.6050*
*τ*_in (CTL)_ [min]	*3.0* ± *1.8*	*3.5* ± *1.7*	*4.0* ± *1.9*	*3.3* ± *1.4*	*2.5* ± *1.6*	*9.7* ± *3.0*	*2.6* ± *1.7*	*1.6* ± *0.9*	*0.8* ± *0.1*	*0.8* ± *0.1*
*τ*_in (AD)_ [min]	*9.1* ± *5.1*	*25.0* ± *7.3*	*23.6* ± *6.5*	*19.6* ± *6.2*	*12.9* ± *4.4*	*13.8* ± *3.4*	*21.9* ± *6.9*	*9.7* ± *4.5*	*2.7* ± *1.1*	*0.7* ± *0.1*
*p-value (out)* =	*0.5503*	*0.7787*	*0.2947*	*0.7097*	*0.4027*	*0.3415*	*0.8662*	*0.2634*	*0.1631*	*0.9691*
*τ*_out (CTL)_ [min]	*77.3* ± *32.5*	*54.3* ± *7.0*	*43.5* ± *8.4*	*69.6* ± *17.9*	*60.5* ± *9.5*	*179.5* ± *20.5*	*56.8* ± *8.6*	*55.8* ± *10.1*	*41.8* ± *6.1*	*34.3* ± *2.8*
*τ*_out (AD)_ [min]	*56.9* ± *3.5*	*56.4* ± *2.6*	*53.0* ± *1.2*	*62.5* ± *3.8*	*71.9* ± *8.7*	*132.5* ± *41.8*	*55.2* ± *3.1*	*92.0* ± *28.3*	*92.0* ± *32.2*	*33.9* ± *8.5*

### Altered glymphatic transport in AD mice is associated with brain pathology at the microscale

**Fig. 6:**
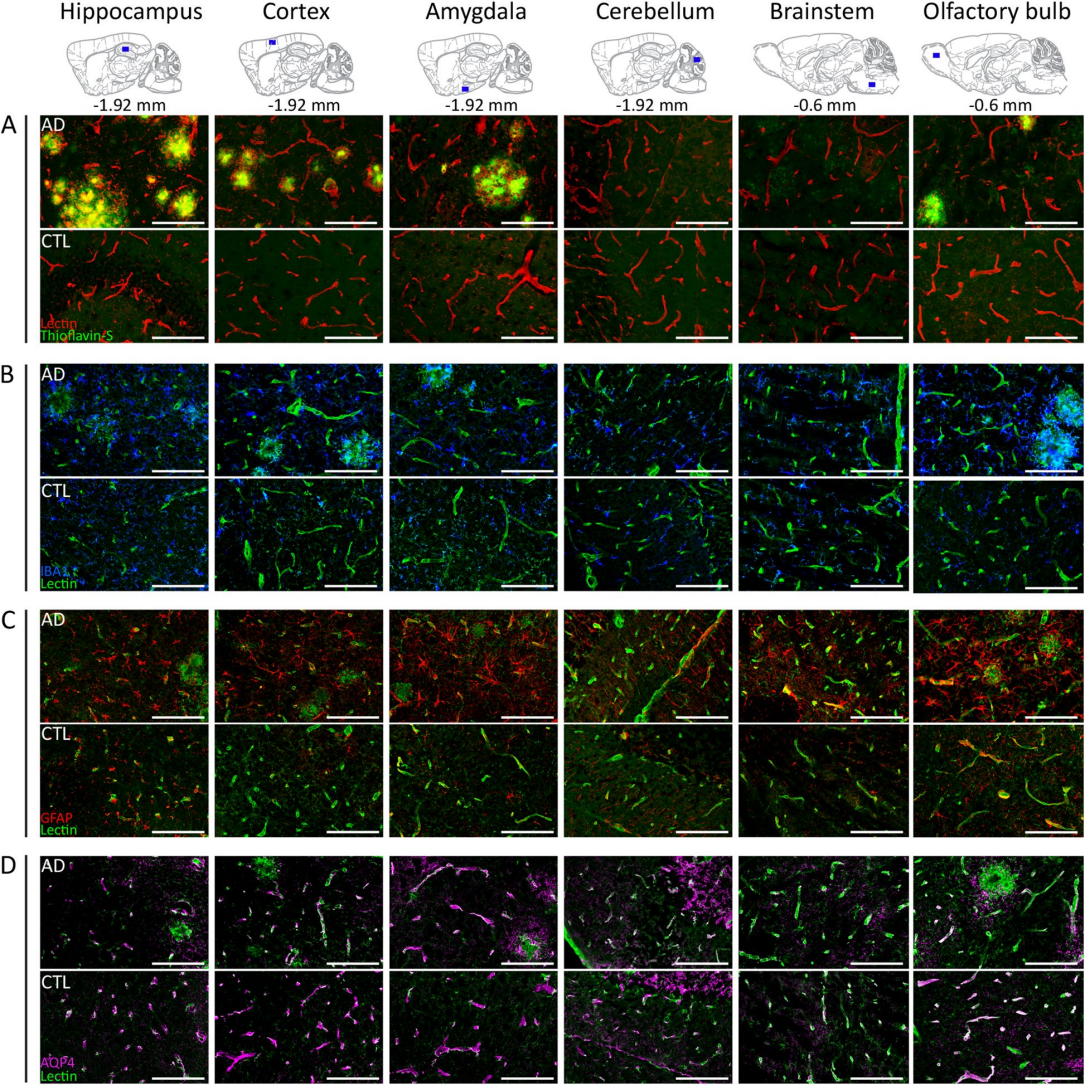
Representative images of amyloid pathology and inflammatory responses in the Tet-Off APP mouse model. Ex vivo evaluation was performed within key brain regions, such as the hippocampus, cortex, amygdala, olfactory bulb, cerebellum and brainstem in Tet-Off APP (AD) and control (CTL) mice. The uppermost panel shows sagittal planes (interaural respectively at − 1.92 mm or − 0.6 mm) from the Paxinos atlas indicating the selected brain slices and area locations. **A** Amyloid plaques (green) stained with Thioflavin-S were detected in brain regions characteristic of the model, such as the hippocampus, the cortical areas and olfactory bulb. The blood vessels were labelled with lectin (tomato—red). **B** Clear evidence of microgliosis within the brain tissue with characteristic large clusters of microglia (Iba-1, blue) observed in AD mice; blood vessels (lectin, green). **C** AD mice showed extensive and wide-spread astrogliosis, astrocytes identified by GFAP immunostaining (red), compared to control littermates even in brain regions devoid of plaques (i.e. brainstem); blood vessels (lectin, green). **D** AQP4 (magenta) immunohistochemical staining patterns along blood vessels (lectin, green). Scale bar, 100 µm for all images

To further clarify the observed differences in the dynamics of brain-fluid circulation in the AD group, we have also performed an ex vivo evaluation by immunohistochemistry to assess microscale alterations in brain regions key to our studies. To this end, we evaluated both the Aβ plaque burden (Thioflavin-S staining) and the inflammatory responses, such as astrogliosis (GFAP staining) and microgliosis (Iba-1 staining) (Fig. 6A–C). Accordingly, a large Aβ plaque burden with characteristic dense-core plaques was observed throughout the brain of AD mice, including the hippocampus, cortex, olfactory bulb and amygdala, with no deposits present in the cerebellum and the brainstem (Fig. 6A), thereby consistent with the previously reported forebrain parenchymal amyloidosis for this Tet-off APP mouse model [42]. In terms of inflammatory processes, microgliosis and extensive wide-spread astrogliosis were clearly present in the brain of AD mice (Fig. 6B, C). Large dense clusters of reactive microglia (blue) with strong signal intensity were found within regions affected by high amyloid deposition (Fig. 6B) such as the cortical areas and olfactory bulb. Astrogliosis (total GFAP signal) was evaluated using LME analysis (see the ‘Methods’ section). We found a statistically significant effect of genotype (LME, *p* = 2.05e − 6) demonstrating decreased astrogliosis in the controls 44.17 (95% CIs: 31.26–57.08) versus the AD mice that in the cortex showed higher GFAP-signal levels of 62.62 (95% CIs: 48.15–77.09). Notably, high levels of astrogliosis were also observed in brain regions devoid of plaques such as the brainstem 62.18 (95% CIs: 56.18–68.17) suggesting the spread of inflammatory responses (for more details on the GFAP-signal LME parameter fit, see Supplementary Table 1). In addition, we assessed fractional vessel coverage by astrocytes and found a significant reduction in AD mice (LME, *p* = 0.01), 0.51 (95% CIs: 0.46–0.56) compared to the controls 0.59 (95% CIs: 0.48–0.69) without much variation across areas (Supplementary Table 2). Last, the vessel density LME model did not show a significant difference (LME, *p* = 0.34) across the AD mice and controls (Supplementary Table 3). Due to low signal intensity and background signal, we did not attempt to quantify AQP4 polarisation but included representative staining results in Fig. 6D that allow a qualitative assessment (Fig. 6D).

## Discussion

Motivated by recent findings linking AD and ageing to CSF circulation and neuronal network dysfunction, we performed this DCE-MRI study to uncover and better understand potential brain-fluid circulation disturbances in a LOAD mimicking mouse model of amyloidosis. To this end, we used 14-month-old Tet-Off APP (AD) mice in which the APP overexpression, and thus Aβ overproduction, started after the early critical phases of postnatal development at the age of 3 months when the mice are considered mature.

As captured by in vivo DCE-MRI, we found clear differences in the distributions of the contrast agent over time (reflecting brain-fluid circulation) in AD mice compared to age-matched control littermates (Figs. 2 and 4). More specifically, glymphatic transport of the Gd-DOTA tracer was significantly altered in AD mice compared to the controls, with the patterns indicating a reduced and redirected glymphatic flow (Fig. 2C). Notably, we observed that the AD group showed (1) slower infiltration, (2) longer time-to-peak and (3) higher accumulation of CSF tracer in caudal regions of the brain proximal to the CM, such as the medulla and the pons (Fig. 3D, E). These changes can be observed directly when examining the time courses and distribution of clusters of voxels with similar profiles in each of the two groups (Fig. 4). In the AD group, the peak intensity for the clusters located in the vicinity of the injection site was reached with a delay compared to the control group. Moreover, the modification of Gd-DOTA circulation was reflected by the differences of the influx time constant *τ*_in_ (see Fig. 5; Tables 1 and 2) displaying longer and slower parenchymal infiltration of CSF tracer for the AD group irrespective of which group was used as a reference for selecting the clusters.

Previously, we reported that the same AD mice showed a large deterioration of global rsfMRI networks, between regions of the default-mode network (DMN) but also in the whole brain [39]. In that study, an alteration of the rsfMRI networks in AD mice was observed initially solely in the DMN at 2 months of APP overexpression—when Aβ plaques were absent—but soluble Aβ was increased by 20-fold. Later, at 7 months of APP overexpression when a wide-spread Aβ plague load and inflammatory responses were detected, we observed further deterioration in the DMN as well as additional alterations in the whole brain [39]. Soluble Aβ is recognised as one of the key contributors in the induction of abnormal neuronal network excitability in AD [50–53]. For instance, a high level of soluble Aβ was reported to affect glutamate uptake at synapses, leading to glutamate spillover and aberrant activation [54, 55]. Thus, soluble Aβ that circulates via the CSF not only can adhere to pre-existing plaques but also lead to synaptic and astrocytic dysfunction, amplifying amyloid pathology and inflammation. To this end, Peng et al. demonstrated deleterious effects of injected soluble Aβ on glymphatic function in mice [15]. Importantly, Han and colleagues demonstrated that the coupling between the global fMRI signal and CSF influx was correlated with AD pathology in humans [32]. Thus, taken together, our findings from both studies are consistent with those results in humans, pointing towards mutual interactions between CSF flow and global brain activity in AD.

Our immunohistochemistry results in the AD mice showed wide-spread amyloidosis throughout several regions including the cerebral cortex, hippocampus, olfactory bulb and amygdala, while the cerebellum and brainstem remained free of Aβ plaque deposits. In addition, we found a wider extent of astrogliosis present beyond the aforementioned areas with Aβ plaque deposits, like the brainstem. Altogether, we reasoned that (i) the high abundance of Aβ plaques across the forebrain of the AD mice might have hindered the normal routes of CSF circulation within the cranial cavity and (ii) that the detrimental effects of Aβ extended far beyond those areas loaded with plaques inducing wide-spread inflammatory and aberrant responses throughout the brain. Previous studies demonstrated that the blockage of normal routes can redirect and/or impair the CSF bulk flow [15, 56, 57]. To this end, Ma and colleagues observed that the CSF tracer was rerouted to the spine and spinal lymphatic clearance pathways after a CM injection in glioma-bearing mice. The researchers concluded that this was due to the blockage of cranial CSF clearance routes [57]. Furthermore, Wang and colleagues showed that CSF flow was halted in the hemisphere affected by multiple microinfarctions and was slowed on the contralateral side [56]. Also, in the context of amyloidosis models mimicking EOAD in humans, the glymphatic transport of injected radiolabelled Aβ and inulin was greatly reduced in old APP/PS1 mice (12–18 months old), as revealed by ex vivo radioactivity [15]. Accordingly, the glymphatic inflow of intracisternally injected ^125^I-Aβ40 and ^14^C-inulin was reduced in APP/PS1 mice (12–13 months old) compared to controls, in alignment with our results in Tet-Off APP mice. The authors further showed that the clearance of both ^125^I-Aβ40 and ^14^C-inulin (intraparenchymal microinjection into frontal cortex) was reduced in APP/PS1 (12–18 months old) at 30 min post-injection, with a greater effect observed for inulin than Aβ40 (twofold vs. 1.2-fold). A direct comparison with our study here that found no significant difference in the efflux time constant of Gd-DOTA is difficult given the differences in the sensitivity of the methods (radioactivity vs. MRI) and experimental setup (e.g. different tracers and injection sites) and anaesthesia protocol (isoflurane vs. ketamine/xylazine). In respect to the latter, it is well known that different anaesthetics and the animal state in general (awake, anesthetised) induce differences in glymphatic transport in rodents [47–49, 58]. To this end, Bechet and colleagues demonstrated that dorsal accumulation and brain-wide glymphatic transport of fluorescent CSF tracers was reduced under isoflurane when compared to ketamine/xylazine; however, the distribution in the cerebellum and hindbrain did not differ statistically between the two anaesthetics [48]. Also, Xue et al. reported that accumulation of Gd-DOTA in the cerebellum and brainstem was largely similar between isoflurane and ketamine/xylazine [49]. Moreover, differences between the models per se could also contribute to the findings. Note that in APP/PS1 mice, the deposition of plaques begins earlier (at 6 weeks of age) and has a wider spread into regions including the cortex, hippocampus, striatum, thalamus, cerebellum, brainstem and vessels [24]. Kim et al. have recently demonstrated in the same APP/PS1 mice that cerebral amyloid angiopathy (CAA) aggravates perivascular clearance impairment not only in old-aged (19–21 months) but also in younger (7–9 months) mice [59]. More specifically, in the studies of Kim and colleagues, vascular amyloid deposition led to a disruption of the arterial basement membrane integrity and morphology changes along with augmented vascular pulsation and decrease in vascular smooth muscle cell coverage detected upon CAA progression. Noteworthy, the authors also reported that areas with higher CSF influx of tracer were those with the lowest Aβ plaque burden (ventral parts). Thus, they suggested that the CAA’s role in arterial function and structure is not only a consequence but also a contributor in the process of observed impaired CSF tracer transport in AD. In addition, recent findings indicate that lymphatic drainage in the dura mater predominantly occurs in the ventral part rather than the dorsal part, that the basal meningeal lymphatic vessels (mLV) are hotspots for the clearance of CSF macromolecules and that both mLV integrity and CSF drainage are severely impaired with ageing [60]. Thus, taken the above, we can conjecture that ageing, which would reduce CSF drainage in both AD and control mice and lower the efficacy of basal mLV in CSF drainage, could have to some extent contributed to the fact that we did not capture differences in efflux across the two groups.

Consistent with our results, alterations in the glymphatic system were also observed in humans. It was shown that glymphatic transport in AD patients was reduced [61] and delayed compared to healthy controls, as demonstrated by a higher signal retention [62]. Furthermore, cognitively affected patients with idiopathic normal pressure hydrocephalus also displayed a delayed clearance of CSF tracer from the entorhinal cortex [63, 64].

Importantly, the glymphatic system decline was also found upon normal ageing [65]. Kress et al. elegantly demonstrated that not only does the glymphatic flow dramatically reduce upon ageing [22], but also CSF production and pressure decrease as well [19, 66]. This reduction in CSF flow is postulated to be linked to an increased vascular stiffness and reduced brain artery pulsation [22, 67–69]. Moreover, ageing was also associated with worse sleep quality [34], which in turn is linked to increased Aβ levels [70]. In fact, sleep disruption is correlated to an increased Aβ deposition [71] and connected to cognitive dysfunction [72].

Noteworthy, during ageing, not only does the glymphatic system undergo deterioration, but impairments are also observed within the interconnected cerebral lymphatic network, which results in severely reduced drainage towards the peripheral lymph nodes (the main exit routes) [73, 74]. Indicatively, studies performed in 5xFAD mice (an EOAD mouse model) showed that the disruption of meningeal lymphatic vessels in younger animals (5–6 months) led to an exacerbation of the disease, as reflected by increased Aβ deposition and reduced extracellular clearance [75]. More recently, the same authors demonstrated the deterioration of the lymphatic vasculature in 13–14 months old mice [73]. These 5xFAD mice rapidly develop amyloid pathology with plaque deposition starting around 2 months and spreading throughout the cortex, hippocampus, thalamus, olfactory bulb, brainstem and the spinal cord, albeit being absent in the cerebellum. In addition, Mentis and collaborators stipulated that APOE4, the major genetic risk factor for LOAD, could affect the meningeal lymphatic vessel functions, leading to premature shrinkage and, subsequently, to a reduced brain-fluid circulation [76, 77]. In future studies, it would be of interest to explore this glymphatic-lymphatic axis in the Tet-Off APP mouse model using more sensitive tools and/or at earlier time points, preceding amyloid deposition. Thus, the absence of significant differences in the efflux time constants *τ*_out_ between AD and control mice in this study may be also related to the above discussed impact of ageing on the vasculature and the efficacy of the (g)lymphatic system in these middle-aged mice.

Furthermore, we conjecture that changes in the glymphatic flow in AD mice may be partly related to changes in astrocytes. In support, we observed extensive astrogliosis, not only in the vicinity of Aβ deposits, but also throughout the brain and in regions devoid of lesions such as the brainstem (Fig. 6C). Astrocytes are believed to be key players in the modulation of CSF-ISF exchange facilitated by AQP4 water channels at the astrocytic end-feet [8, 17, 58, 68]. Deletion of AQP4 in middle-aged APP/PS1 mice (12 months old) increased Aβ accumulation, vascular amyloidosis and aggravated cognitive deficits [26]. The perivascular localisation and expression of AQP4, required for efficient CSF-ISF exchange, was shown to decline with age [22], and in AD patients, AQP4 polarisation was associated with disease stage [28]. In addition, the extent of vascular amyloidosis in EOAD murine models was closely correlated with astrocyte polarisation [29, 78]. More recently, Simon et al. demonstrated that reduced frontal cortical perivascular AQP4 localisation is correlated with local Aβ and p-tau pathology, as well as cognitive decline on early stages of AD [79]. Interestingly, the authors reported the emergence of a dispersed, cortical astrocyte population that showed AQP4 upregulation and a ‘patchy’ pattern of AQP4 immunoreactivity. This upregulation can be possibly linked with astrogliosis. Here, we have also observed wide-spread astrogliosis and changes in vessel coverage by astrocytes in our AD mice, which possibly contributed to impaired CSF-ISF exchange. In addition, in Fig. 6D, we present representative images of immunofluorescent labelling of AQP4 that can provide qualitative visualisation in AD and control mice (Fig. 6D). Quantitative estimation of perivascular AQP4 polarisation would require a larger sample size and confocal images that were not available in our study. Moreover, astrogliosis is a process by which astrocytes react to different forms of neuropathology like in the case of an Aβ insult [4, 78]. Thus, while healthy astrocytes play a vital role in the neuro-glial-vascular unit by connecting the vasculature to neurons to ensure neuronal communication and sufficiency in energy demands [80], astrogliosis contributes to AD pathology by impairments in astrocytic normal function [4]. To this end, the loss of astrocyte polarisation was suggested to be a consequence rather than a cause of Aβ deposition with an observed impairment of gliovascular coupling [78]. In line with this, we have previously demonstrated the toxic effects of soluble Aβ on the rsfMRI neuronal networks preceding Aβ plaque deposition and reactive astrogliosis [39]. Furthermore, astrocytes also contribute to maintaining glutamate homeostasis in the central nervous system [81, 82]. Thus, changes in astrocytes can also mediate neuronal excitotoxicity via excessive [Ca2 +]-mediated glutamate release, the excess of which affects synaptic function, but also potentially exerts modulatory effects on glymphatic function as suggested recently [83].

While it is still impossible to establish to what extent the disruption in global brain activity and CSF circulation plays a role in the initiation of AD pathology, we conjecture that both amyloid accumulation, astrogliosis and altered brain-fluid dynamics contribute to the overall neurodegeneration. Thus, we suggest that future theranostic approaches should jointly consider these deficits rather than focusing on single aspects. This becomes particularly important in view of recent exciting studies that indicated to possible cognitive improvement by decreasing Aβ load either via combining halted APP overexpression and immunotherapy in Tet-Off APP mice [84] or using targeted enhancement of (g)lymphatic CSF-lymphatic clearance in 5xFAD mice [73, 85, 86].

## Conclusions

To conclude, this study used a mature-onset Tet-Off APP mouse model of amyloidosis, which resembles LOAD in humans, to uncover impairments in brain-fluid circulation at advanced stages of pathology. The results, together with previous findings of functional network dysfunction from our group and others, point towards a complex interplay between the effects of Αβ and dysfunctions in brain clearance mechanisms in AD. To better understand the link between Αβ and brain-fluid circulation, it would be very interesting to further extend these studies by choosing different mouse-age regimes for APP expression, in analogy to the work of Jankowsky and colleagues [87]. Alternatively, other transgenic models overproducing Aβ with APP expressed at physiological levels could be also used. Future studies of the glymphatic-lymphatic axis in relation to network dysfunctions in neurodegenerative disorders both at the whole brain scale by MRI as well as at the microscale by immunohistochemistry and other techniques may provide further insight into the cadence of dysfunction related to amyloid buildup and cognitive dysfunction. Such studies will also highlight the translational potential of alternative targets for the development of non-Aβ-targeted therapeutic strategies.

## Supplementary Information

**Additional file 1. **Supplementary data, figure and tables.

## Data Availability

Raw data and images are available upon reasonable request from the corresponding authors.

## Abbreviations

Aβ: Amyloid-beta
AD: Alzheimer’s disease
ANTs: Advanced Normalisation Tools
APP: Amyloid precursor protein
AQP4: Aquaporin-4
AUC: Area under the curve
CamKIIα: Calmodulin-dependent protein kinase type II alpha chain
CB: Cerebellum
CM: Cisterna magna
CSF: Cerebrospinal fluid
CTL: Control
DCE-MRI: Dynamic contrast-enhanced magnetic resonance imaging
DMN: Default-mode (like) network
DOX: Doxycycline
EOAD: Early onset of Alzheimer’s disease
Gcv: Cerebral vein of Galen
Gd: Gadolinium
Gd-DOTA: Gadoteric acid
GS: Glymphatic system
HC: Hippocampus
ISF: Interstitial fluid
LOAD: Late onset of Alzheimer’s disease
MREG: Magnetic resonance encephalography
NONE: Non-injected
NREM: Non-rapid eye movement
NTg: Non-transgene carrier
OB: Olfactory bulb
OlfA: Olfactory artery
PCA: Principal component analysis
ROI: Region-of-interest
Rrhv: Rostral rhinal vein
rsfMRI: Resting-state functional magnetic resonance imaging
SAL: Saline
Sss: Superior sagittal sinus
tetO: Tetracycline-responsive
Trs: Transverse sinus
tTA: Tetracycline transactivator
