# Supplementary material for: Altered dynamics of glymphatic flow in a mature-onset Tet-off APP mouse model of amyloidosis
Source: Alzheimers Res Ther. 2023 Jan 28;15:23. doi: 10.1186/s13195-023-01175-z (PMC9883946; doi:10.1186/s13195-023-01175-z)
Supplement: Supplementary file 1 — Additional file 1. Supplementary data, figure and tables. [file 13195_2023_1175_MOESM1_ESM.pdf]

Supplementary Material

Title: Altered dynamics of glymphatic flow in a mature-onset Tet-off APP mouse model of amyloidosis

Author list: Inès R.H. Ben-Nejma[1#*], Aneta J. Keliris[1#*], Verdi Vanreusel[1,3], Peter Ponsaerts[2], Annemie Van der Linden[1,4], Georgios A. Keliris[1,4,5*]

Author affiliations:

[1]Bio-Imaging Lab, University of Antwerp, Universiteitsplein 1, 2610 Wilrijk, Antwerp, Belgium.

[2]Laboratory of Experimental Hematology, Vaccine and Infectious Disease Institute (Vaxinfectio), University of Antwerp, Universiteitsplein 1, 2610 Wilrijk, Antwerp, Belgium.

[3]Research in dosimetric applications, SCK CEN, Boeretang 200, 2400 Mol, Antwerp, Belgium.

[4]µNEURO Research Centre of Excellence, University of Antwerp, Antwerp, Belgium.

[5]Institute of Computer Science, Foundation for Research and Technology – Hellas (FORTH), Heraklion, Crete, Greece

Voxel-based analysis reflects differences in the distribution of Gd-DOTA across groups.

To assess the differences in the spatial distribution of the Gd-DOTA between the CTL and AD groups, voxel-based analysis (VBA) was performed at four time-points after injection (30, 60, 90, 120 min). To this end, a two-sample *t*-test was performed on a voxel-by-voxel basis comparing the percent signal change from baseline across the CTL and AD groups. No statistical significance was observed after multiple comparison correction, but Additional Figure 1 presents uncorrected statistics ($p<0.05$, uncorrected) that indicate a general agreement with our ROI-based and cluster-based analyses. Specifically, percent signal change was higher in the AD group in areas nearby the injection cite (pons, medulla; orange/yellow) as also observed in other analyses. In addition, VBA revealed signal decreases in AD relative to CTL in caudal areas close to the superior sagittal and transverse sinuses (blue) but note that signal intensity in those areas was low and within the variability observed in the non-injected groups and thus it is difficult to make robust conclusions.

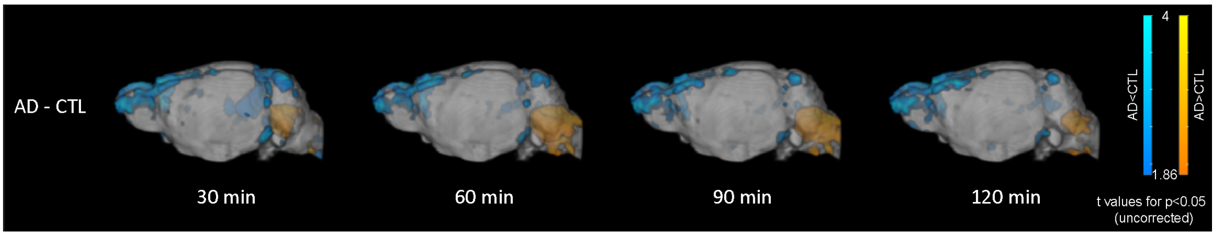

Additional Figure 1. Voxel-based analysis (VBA). *The signal intensity of CTL and AD mice were compared at four time-points after injection (30, 60, 90, 120 min). The AD group demonstrated stronger signal intensity in regions adjacent to the infusion spot (orange/yellow) in all time points and decreased signal intensity in areas proximal to the superior sagittal and transverse sinuses (blue). The colour scales indicate T-statistic values, with orange/yellow representing the voxels significantly higher in the AD group and blue/cyan representing the voxels significantly higher in the CTL group (p<0.05, uncorrected).*

Supplementary Statistics

***Supplementary Table 1**: Linear Mixed-Effects model analysis for total GFAP signal*

```
Linear mixed-effects model fit by ML

Model information:
    Number of observations             72
    Fixed effects coefficients          4
    Random effects coefficients         6
    Covariance parameters               2

Formula:
    Mean ~ 1 + Brain_area + mouse_type + (1 | mouse_id)

Model fit statistics:
    AIC       BIC       LogLikelihood    Deviance
    603.48    617.14    -295.74          591.48
Fixed effects coefficients (95% CIs):
    Name                           Estimate    SE        tStat      DF    pValue        Lower      Upper
    {'(Intercept)'        }         62.176     3.0028     20.706    68     4.486e-31     56.184     68.168
    {'Brain_area_CTX'     }        0.44392     4.2466    0.10453    68       0.91705      -8.03     8.9179
    {'Brain_area_Amygdala'}        -18.874     4.2466    -4.4444    68    3.3347e-05    -27.348      -10.4
    {'mouse_type_WT'      }        -18.005     3.4673    -5.1926    68    2.0482e-06    -24.924    -11.086
Random effects covariance parameters (95% CIs):
Group: mouse_id (6 Levels)
    Name1                  Name2                  Type         Estimate      Lower    Upper
    {'(Intercept)'}        {'(Intercept)'}        {'std'}      1.6332e-15    NaN      NaN
Group: Error
    Name               Estimate    Lower     Upper
    {'Res Std'}        14.711      12.494    17.321
```

***Supplementary Table 2**: Linear Mixed-Effects model analysis for vessel coverage*

```
Linear mixed-effects model fit by ML

Model information:
    Number of observations             73
    Fixed effects coefficients          4
    Random effects coefficients         6
    Covariance parameters               2

Formula:
    coverage ~ 1 + group + area + (1 | id)

Model fit statistics:
    AIC        BIC        LogLikelihood    Deviance
    -94.209    -80.467    53.105           -106.21
Fixed effects coefficients (95% CIs):
    Name                   Estimate     SE          tStat     DF    pValue        Lower       Upper
    {'(Intercept)'  }        0.51311    0.023811    21.549    69    2.3339e-32     0.46561      0.56061
    {'group_WT'     }       0.071953    0.027375    2.6284    69      0.010565    0.017341      0.12656
    {'area_CTX'     }      -0.059394    0.033158   -1.7913    69      0.077636    -0.12554    0.0067536
    {'area_Amygdala'}      -0.056074    0.033747   -1.6616    69       0.10113     -0.1234      0.01125
Random effects covariance parameters (95% CIs):
Group: id (6 Levels)
    Name1                  Name2                  Type         Estimate      Lower    Upper
    {'(Intercept)'}        {'(Intercept)'}        {'std'}      2.5958e-17    NaN      NaN
Group: Error
    Name               Estimate    Lower       Upper
    {'Res Std'}        0.1169      0.099399    0.13749
```

***Supplementary Table 3*: Linear Mixed-Effects model analysis for vascular density**

```
Linear mixed-effects model fit by ML

Model information:
    Number of observations             18
    Fixed effects coefficients          2
    Random effects coefficients         6
    Covariance parameters               2

Formula:
    x_Area ~ 1 + mouse_type + (1 | mouse_id)

Model fit statistics:
    AIC       BIC       LogLikelihood    Deviance
    60.651    64.212    -26.325          52.651
Fixed effects coefficients (95% CIs):
    Name                     Estimate    SE         tStat      DF    pValue        Lower       Upper
    {'(Intercept)'  }         4.7046     0.50422     9.3303    16    7.1419e-08      3.6357    5.7735
    {'mouse_type_WT'}        0.69578     0.71308    0.97574    16       0.34372    -0.81588    2.2074
Random effects covariance parameters (95% CIs):
Group: mouse_id (6 Levels)
    Name1                  Name2                  Type           Estimate    Lower      Upper
    {'(Intercept)'}        {'(Intercept)'}        {'std'}        0.71525     0.30083    1.7006
Group: Error
    Name               Estimate    Lower      Upper
    {'Res Std'}        0.86799     0.58179    1.295
```
